# The combined use of platinum nanoparticles and hydrogen molecules induces caspase-dependent apoptosis

**DOI:** 10.1186/1753-6561-7-S6-P109

**Published:** 2013-12-04

**Authors:** Takeki Hamasaki, Tomoya Kinjyo, Hidekazu Nakanishi, Kiichiro Teruya, Sigeru Kabayama, Sanetaka Shirahata

**Affiliations:** 1 Department of Bioscience and Bioengineering, Faculty of Agriculture, Kyushu University, 6-10-1 Hakozaki, Higashi-ku, Fukuoka 812-8581, Japan; 2 Graduate School of Systems Life Sciences, Kyushu University, 6-10-1 Hakozaki, Higashi-ku, Fukuoka 812-8581, Japan; 3 Nihon Trim Co. LTD., 34-8-1 Ooyodonaka, Kita-ku, Osaka 531-0076, Japan

## 

We previously reported electrochemically reduced water (ERW), produced near the cathode by electrolysis, exhibits reductive activity. We also revealed that ERW contains Pt nanoparticles (Pt nps) derived from Pt-coated titanium electrodes in addition to high concentration of dissolved molecular hydrogen (H_2_) by in vitro assay, and Pt nps exhibit powerful ROS scavenger activity and catalysis activity converting H_2 _to active hydrogen. Our study investigates apoptosis inducibility of H_2 _and synthesized Pt nps on human promyelocytic leukaemia HL60 cells. Human promyelocytic leukaemia cells (HL60) were cultured in RPMI 1640 medium supplemented with 10% FBS, 2.0 mM l-glutamine, 100 U/ml penicillin and 100 U/ml streptomycin. Cultures were incubated in an atmosphere of 75%(v/v) H_2_/20%(v/v) O_2_/5%(v/v) CO_2_, 75%(v/v) He/20%(v/v) O_2_/5%(v/v) CO_2 _atmosphere or 75%(v/v) N2/20%(v/v) O_2_/5%(v/v) CO_2 _atmosphere for 12-48 hr after incubated with Pt nps for 2 h. Untreated cultures were included as controls. Cytotoxicity was determined by cell-counter. Apoptosis pathway of HL60 cells was investigated by Sub G-1 assay.

Growth suppression was not observed when cells were treated with Pt nps or H_2 _only. Analysis of cell cycle and activity of caspase-3 suggested that combination use of both Pt nps and H_2 _induced apoptosis in HL60 cells. Our caspase activity experimentation suggests that apoptosis was caused via caspase-8 activation. These results suggested that atomic hydrogen from H_2 _induces caspase-8 dependent apoptosis. The cytotoxicity was not detected in Pt nps or H_2 _separately treated cells. Apoptosis was determined only when cells were treated with both Pt nps and H_2_, suggesaspase-8 dependent apoptosis was caused by atomic hydrogen produced from H_2 _by catalyst activity of Pt nps.

